# Dehydrochlorination of PCDDs on SWCN-Supported Ni_10_ and Ni_13_ Clusters, a DFT Study

**DOI:** 10.3390/molecules27165074

**Published:** 2022-08-10

**Authors:** Silvia González, Martha Porras, Arianna Jimbo, Cesar H. Zambrano

**Affiliations:** 1 Departamento de Química, Facultad de Ciencias Exactas y Naturales, Universidad Técnica Particular de Loja, San Cayetano Alto, Calle Marcelino Champagnat s/n, Loja 110101, Ecuador; 2 Universidad Técnica de Machala, Av. Panamericana Km. 5 1/2 Vía a Pasaje, Machala 170526, Ecuador; 3 Departamento de Ingeniería Química, Universidad San Francisco de Quito, Pampite y Robles s/n Cumbayá, Quito 170901, Ecuador

**Keywords:** nanotubes, dibenzo-p-dioxins, DFT, nickel, clusters, dehydrochlorination

## Abstract

Polychlorinated dibenzo-p-dioxins (PCDDs) are known to be a group of compounds of high toxicity for animals and, particularly, for humans. Given that the most common method to destroy these compounds is by high-temperature combustion, finding other routes to render them less toxic is of paramount importance. Taking advantage of the physisorption properties of nanotubes, we studied the reactions of atomic hydrogen on physisorbed PCDDs using DFT; likewise, we investigated the reaction of molecular hydrogen on PCDDs aided by Ni_10_ and Ni_13_ clusters adsorbed on single-wall carbon nanotubes. Because dihydrogen is an easily accessible reactant, we found these reactions to be quite relevant as dehydrohalogenation methods to address PCDD toxicity.

## 1. Introduction

Among the toxic chemical compounds of major concern to human and animal wellbeing, dioxins, which comprise polychlorinated dibenzo (PCDDs) derivatives, as well as dibenzofuran analogs (PCDFs) are among the most dangerous compounds known to man [1]. One compound of particular interest is the 2,3,7,8-tetrachlorodibenzodioxin, also known as TCDD, which is the most dangerous one reported [2]. The toxicity of these compounds arises not only because of their environmental resilience, meaning their high stability and long half-life which may exceed one decade [3], but mainly because they interfere with important enzymatic processes in the body. A relevant effect of these compounds is their interaction with a specific intracellular protein, the aryl hydrocarbon or Ah receptor, which is a transcription enhancer that interacts with a number of regulatory proteins (hormones) and regulates gene expression, immunity, stem cell maintenance and cellular differentiation [1,4,5]. However, the harmfulness and bioaccumulation of dioxins in the environment and in organisms, including human beings, are not yet totally defined and clarified [6].

In spite of the fact that dioxins are manufactured in very limited quantities and for research purposes only, the larger amounts found in soils, water sources, animals’ foodstuff and in the air are the result of industrial processes such as the incineration of municipal waste, bleaching of paper pulp and pesticide manufacturing, among others [7,8,9].

Many methods and studies have been developed for the elimination and destruction of dioxins [9]. Although the most effective is still incineration, other methodologies include supercritical water oxidation, hydrothermal decomposition, ionizing radiation processes, and metal smelting treatment, among others [10,11,12,13]. Among the studies directed towards dioxin extraction, several novel approaches have appeared in the literature, some of them involving graphene as an adsorbent, whereas others are about adsorption onto carbon nanotubes. Interestingly enough, it was shown that the electronic properties of single-wall and multi-wall carbon nanotubes (SWCNs and MWCNs, respectively) are potentially useful in removing dioxins via an adsorption process much stronger than that initially conceived. Furthermore, SWCNs doped with the main group and transition metals exhibit especially strong interactions [14,15].

Carbon nanotubes have been proposed as sorbent for several adsorbates [16] and dioxin removal [17], and recent DFT calculations [18] indicate that the TCDD molecule is weakly bound (physisorbed) to the outer surface of a pure carbon nanotube with an energy of −0.52 eV [15], whereas greater energy can be found for doped carbon nanotubes [19]. Additionally, several metals have been used experimentally as heterogeneous catalysts supported on oxides [20,21] and carbon nanotubes for reducing dioxin concentration [22].

The ability of nanotubes to capture hydrogen is valuable for confining it and making it available for a variety of uses [23]. Although the precise interaction of hydrogen adsorption on carbon nanotubes is not profoundly described because it could be physisorption or chemisorption [24], more authors tend to affirm that the H_2_ molecule is physisorbed on a carbon nanotube [25,26,27]. Thus, it has been reported that in order to activate the chemisorption in this system [28,29], it must be modified [30] or functionalized, i.e., by doping. However, atomic hydrogen does adsorb spontaneously on carbon nanotubes [31]. Beheshtian et al. [32] calculated the atomic H-adsorption on a pristine carbon nanotube and a 1,3-cyclohexadiene functionalized carbon nanotube, and concluded that both processes are exothermic. The dissociation energy for the H_2_ molecule on the pure and functionalized carbon nanotube was calculated, and it was found to be −1.00 eV and −1.55 eV, respectively. The activation energy was 0.19 eV greater for the functionalized carbon nanotube, which showed a value of 3.70 eV.

Alonso et al. [25] studied the adsorption of molecular and atomic hydrogen on carbon nanotubes via static and dynamical calculations. They found that physisorption is possible outside a single wall carbon nanotube for both a H atom and also for a H_2_ molecule. For the H atom, it was reported that it adsorbs with energies ranging from −0.9 to −1.63 eV, whereas the interaction energy between a SWCN and a H_2_ molecule is about 0.07 eV.

Several metallic clusters have been employed as models to study the catalytic and adsorption properties of metals [33,34,35]. In particular, Ni clusters were used to investigate molecular dissociation [36,37,38] and other reactions [39,40]; specifically, some experimental works about the interaction of hydrogen and Ni clusters [41] report that the presence of Ni decreases the barrier for H elimination from isopropanol, and that Ni clusters in ternary composites can generate a high photocatalytic hydrogen production, among other effects. Felício-Sousa explained the high adsorption energy found for H_2_ on Ni_13_, with respect to other metals, by the enhancement of the sp–d hybridization due to the shift of the d-states towards the highest occupied molecular orbital (HOMO) [42].

Carbon nanotubes have been functionalized with metals, improving their adsorption characteristics, as shown by Verdinelli et al. [43], who used a Ru atom anchored on a carbon nanotube to study the adsorption properties of H and H_2_, and demonstrated that the adsorption of H_2_ on a Ru-anchored carbon nanotube is more dissociative (E_ads_ = −0.697 eV) compared to a pure carbon nanotube [43,44]. Singh et al. studied the effects of a Ni_13_ cluster on a carbon nanotube for CO detection [44]. They found that the most favorable attachment site is right on the hollow site of a six-membered ring of the carbon nanotube, similar to what was reported by Verdinelli for a single Ru atom. The cluster exhibited an adsorbed binding energy of 2.40 eV.

Related to the aforementioned reports, the behavior of graphene flakes with nickel nanoclusters or hydrogen molecules has been studied via molecular dynamics simulations; the results indicate that graphene flakes can be an excellent container for a metal nanocluster since graphene can easily trap it, but the amount of hydrogen stored is low [45]. This demonstrates an affinity of the aromatic 2-D ring sheets for metal clusters. Another published work indicated that the adsorption energy per H atom is larger for Pd/Ni-functionalized nanotubes than for pure Pd-functionalized carbon nanotubes [46].

Zhang et al. [47] carried out an ab initio mechanistic investigation of H_2_ with pure and N-doped carbon nanotubes. They found that adsorbed hydrogen atoms can act as an autocatalyst for further dissociative adsorption of hydrogen molecules. Doping the nanotube with nitrogen considerably altered the catalytic effects of the carbon nanotube for hydrogen dissociative adsorption. The dissociative adsorption of hydrogen was greatly enhanced, with the barrier substantially reduced to 0.9 eV.

Adsorption binding energies of nickel-doped SWCNs were calculated by Seenithurai et al. [48], and they reported that that the H_2_ binding energies fluctuated until passivation via H-atom addition on the metal (hydride formation) occurred. It was found that the nickel atom preferred a C-C bridge position instead of the position at the center of the hexagon. The H_2_ binding energies reported revealed that desorption would take place above room temperature in Ni-doped (5,0) carbon nanotubes.

Several research works have been devoted to the hydrochlorination of molecules on surfaces to increase these values, and the dehydrochlorination of chlorinated organic compounds to solve environmental problems [49,50]. Fagan et al. studied the various possible configuration interactions between TCDD and a pristine carbon nanotube, finding that the lowest energy is obtained when a TCDD molecule is aligned parallel to the carbon nanotube at −0.77 eV. Furthermore, these investigators reported that a vacancy-defective carbon nanotube exhibited the lowest interaction energy at −1.21 eV [15]. In a more recent investigation, the adsorption energy of the same TCDD molecule was calculated for pure and Al-doped carbon nanotubes, and it was indicated that the binding energy rises to −0.85 eV when TCDD binds to the doped carbon nanotube [19]. Experimentally, several metals have been used as heterogeneous catalysts supported on oxides [20,21] and carbon nanotubes for reducing dioxin concentration [22]; additionally, homogeneous catalysts have been employed for the dechlorination of TCDD [51].

Considering the aforementioned findings, in this study we assess the feasibility of partial polychlorodioxin reduction through a dehydrochlorination mechanism using a computational theoretical approach involving molecular hydrogen, nickel clusters and SWCNs as participating reactants.

## 2. Results and Discussion

In order to chemically render dioxins less harmful, we explored the reaction of hydrogen with dioxins supported by a SWCN and nickel clusters. As it will be shown in the next subsections, dehydrochlorination appears to be possible; however, since dihydrogen dissociation on the carbon nanotube surface is not facile [32,47,52], we conceived the dissociation of molecular hydrogen on nickel clusters prior to the atomic hydrogen reaction with dioxins.

In the next section, we describe the dissociation pathway of dihydrogen in a vacuum as a first and comparison step to the dissociation on surfaces of nickel clusters.

### 2.1. Free H_2_ Dissociation

**Figure 1:**
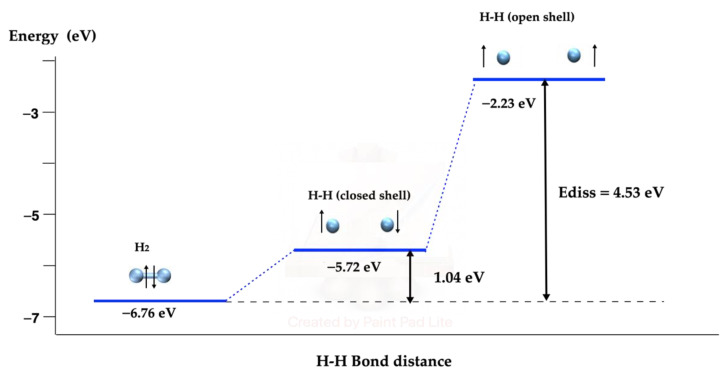
H_2_ dissociation pathway in vacuum. The blue spheres represent H atoms.

The H_2_ molecular dissociation pathway in a vacuum, shown in Figure 1, was calculated and compared with the dissociation pathway in metallic Ni clusters (Section 2.3). Although this reaction pathway in a vacuum and on metallic systems, especially in nickel [53,54,55,56,57,58,59,60,61,62,63,64,65,66,67,68], has been studied extensively, we included it as a part of the process for the dechlorination of dioxins. The molecular dissociation energy (4.53 eV) compares nicely with the experimental data [69]. The closed-shell state is shown as an intermediate; it is well known that the most stable state of the hydrogen molecule is a singlet closed-shell one, whereas the preferred state of the two separated hydrogen atoms is an open-shell state. We note that with both the PBE-GGA functional and the semiempirical GGA-type density functional with dispersion correction, proposed by Grimme [70], the dissociation energy of the hydrogen molecule is the same.

The calculated H_2_ atomic distance was found to be 0.75 Å and the vibrational frequency was 4470 cm^−1^, attributed to the experimental IR data of the stretching vibrational state, reported at 4162 cm^−1^ [71].

### 2.2. H_2_ Dissociation on a SWCN

We calculated the adsorption and dissociation of H_2_ on a SWCN, according to previous studies [25,31,32], using the GGA-DFT approach with dispersion. We found that the interaction between the molecule and the carbon nanotube model is weak, involving 0.54 eV, with no relaxation of the molecular bond, and the distance between the H_2_ molecule and the nearest carbon atoms of the nanotube is 3.1 Å; this distance is very long and confirms the weak interaction between the H_2_ molecule and the SWCN model, which is in agreement with Alonso et al. [25] and their study about the interaction of molecular and atomic hydrogen in carbon nanotubes.

To calculate the adsorption energy (E_adsH2_), the following expression was employed:

E_adsH2_ = E_H2/SWCN_ − E_H2_ − E_SWCN_

where E_H2/SWCN_ is the energy of the molecule close to the SWCN, E_H2_ is the energy of the H_2_ molecule and E_SWCN_ is the energy of the carbon nanotube.

On the other hand, the interaction between the two H atoms and the SWCN is favorable, the adsorption energy (E_adsH-H_) is −1.55 eV, and the distance between both atoms is 5.2 Å; however, the distance from the H atom to the nearest C atom of the nanotube is very short, being 1.2 Å. The adsorption energy was calculated with this expression:

E_adsH-H_ = E_H-H/SWCN_ − E_H-H_ − E_SWCN_

where E_H-H/SWCN_ is the energy of the two H atoms interacting with the SWCN and E_H-H_ is the energy of the two H atoms.

Finally, the energy dissociation (E_diss_) of H_2_ on the SWCN was calculated as the difference between the energy of interaction between the two H atoms near the SWCN (E_adsH-H_) as the final state and the adsorption energy of the H_2_ molecule on the SWCN (E_adsH2_) as the initial state:

E_diss_ = E_adsH-H_ − E_adsH2_

**Figure 2:**
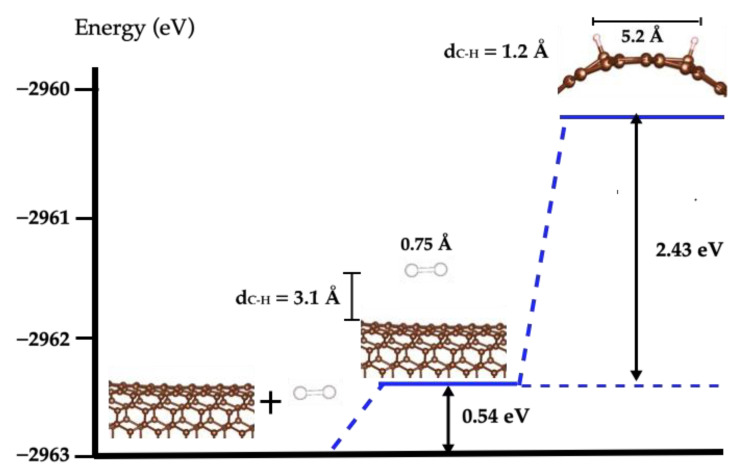
H_2_ dissociation pathway in SWCN. The blue line marks the H_2_ dissociation pathway. The brown spheres represent C atoms and white spheres are H atoms. The origin of the energy scale is the sum of the energy values of the isolated molecule and the bare SWCN.

Additionally, in spite of the molecular dissociation of adsorbed H_2_ not being exothermic and the interaction energy being 2.43 eV, this value is lower than that in a vacuum (4.53 eV, as it was mentioned in the previous section), which means that the SWCN favors the molecular dissociation of H_2_; finally, the distance between the hydrogen atoms is 5.2 Å and Figure 2 illustrates the energy values and the H-H bond length of these states.

### 2.3. H_2_ Dissociation on Ni Metallic Clusters

**Figure 3:**
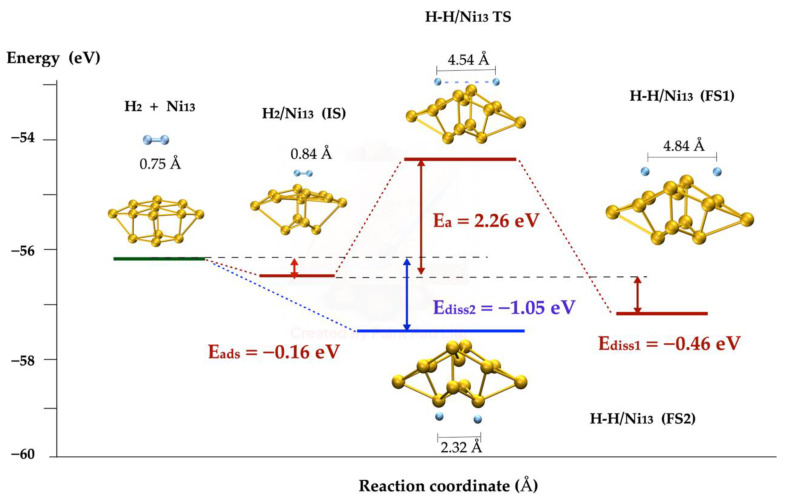
H_2_ dissociation pathways on Ni_13_ cluster. The blue lines mark the spontaneous pathway and red lines correspond to the activated one. The blue spheres represent H atoms and yellow spheres are Ni atoms.

The interaction of the H_2_ molecule and the Ni_13_ cluster is represented in Figure 3. The H_2_ molecule approached the two faces of the cluster. The Ni cluster model is a pyramid composed of nine Ni atoms in its base and four Ni atoms at the top. In the base of the metallic cluster (this pathway is distinguished with the red color in Figure 3), the molecule was adsorbed with −0.16 eV as the physisorption energy, and 12% of the bond distance was relaxed with respect to the 0.75 Å of the isolated molecule. This state was considered as the initial point of the molecular dissociation pathway calculated using the NEB method [72] and the transition state structure was tuned using the dimer method [73], both implemented in the VASP code [74,75,76]. The barrier energy corresponding to the located late transition state (TS in Figure 3) was 2.26 eV. According to the obtained results, the H_2_ dissociation on the Ni_13_ is exothermic because the energy difference between the dissociated state or final state (symbolized as FS1 in Figure 2) and adsorbed state or initial state (IS) was −0.46 eV. On the other hand, the interaction between the molecular hydrogen and the top of the pyramid (the blue line indicates this pathway) leads to the exothermic breakup of the H-H molecular bond involving −1.05 eV; FS2 is the final state of the interaction, with a H-H bond length of 2.32 Å.

**Figure 4:**
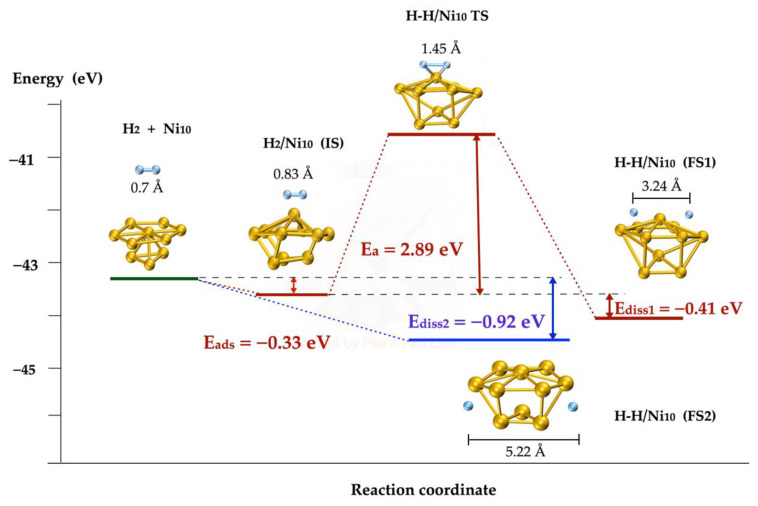
H_2_ dissociation pathways on Ni_10_ metallic cluster. The blue lines mark the spontaneous pathway and red lines correspond to the activated one. The blue spheres represent H atoms and yellow spheres are Ni atoms.

Additionally, the H_2_ dissociation on Ni was tested using a different cluster with 10 atoms, with similar energy results in spite of the differences of the geometric structure of the cluster and of the transition state, as is shown in Figure 4. Here, the exothermic process is evident, with a dissociation energy of −0.41 eV. According to the results obtained, the molecule was dissociated spontaneously on the top of both metallic clusters (Ni_13_ and Ni_10_); on one hand, at the top of the cluster, the energy values were −1.05 eV and −0.92 eV, respectively (the difference between both values is 0.13 eV); on the other hand, on the basis of the dissociation not being spontaneous, the activation energy was 2.26 eV and 2.89 eV, respectively. Hence, both metallic clusters may be used indifferently; therefore, only the Ni_13_ cluster was chosen to be included in the nanotube to continue studying its reaction.

Our results are in agreement with the literature [30,37], which affirms that atomic hydrogen can be readily available when molecular hydrogen is placed in an environment where metal clusters are present, and in our case, this could also be applied for nickel clusters.

### 2.4. H_2_ Dissociation on SWCN Model Functionalized with Ni

**Figure 5:**
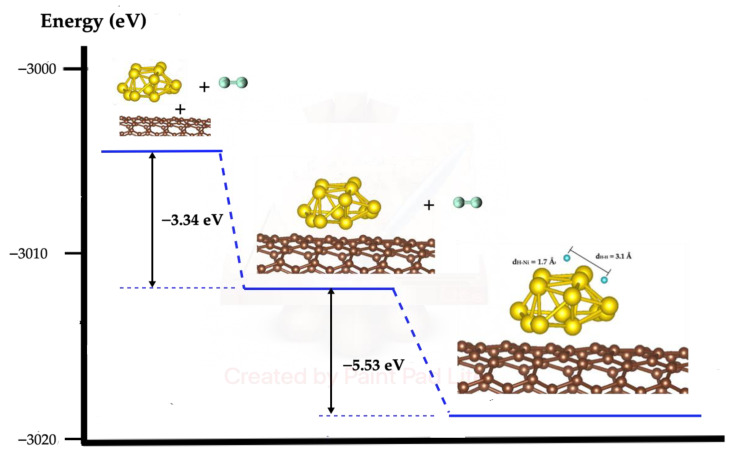
H_2_ dissociation on Ni_13_ metallic cluster adsorbed on SWCN. The yellow spheres indicate Ni atoms, brown spheres represent C atoms and small blue spheres are H atoms.

According to the results described in Section 2.3, the dissociation of the H_2_ molecule is more favorable on the top than on the base of the metal cluster; although the dissociation at the top is spontaneous, at the base it has an activation energy of more than 2 eV, as illustrated in Figure 3 and Figure 4. For the study of the dissociation of the molecule H_2_ on the Ni cluster deposited on the SWCN, only the interaction of the molecule on the top of the cluster was considered, as shown in Figure 5.

The results indicate that, similarly to how the H_2_ molecule is spontaneously dissociated in the isolated nickel metal cluster, the dissociation also occurs spontaneously in the case of the adsorbed cluster on the nanotube, where the calculated value of the dissociation energy is −5.53 eV; the distance between both hydrogen atoms is 3.1 Å and the distance from each H atom to the nearest Ni atom is around 1.7 Å. A brief annotation about the interaction energy between the Ni metallic cluster and the nanotube of −3.34 eV, which indicates that it is energetically favorable, and the distance between Ni atoms of the metallic cluster and the nearest carbon atoms of the SWCN is around 2 Å.

### 2.5. Dehydrochlorination of Polychlorodioxins on a SWCN

The aforementioned evidence encouraged our looking into the possible dehydrohalogenation of dioxins on SWCNs. For this purpose, we built an armchair (8,8) single-wall carbon nanotube and a number of dioxins, as listed in Table 1. To identify each PCCD molecule, abbreviations were assigned to each one as shown in Table 1.

Construction of the dioxin molecules on the surface of the carbon nanotube revealed that the octachlorodibenzodioxin exhibited a physisorption energy of −0.6 eV, and the heptachlorodibenzodioxin of −0.41 eV, whereas the energy associated with the other dibenzodioxins ranged from −0.9 to −1.0 eV. It is conceived that this difference in energies is due to the greater number of chlorine atoms in the former, which makes the SWCN–dioxin interaction less favorable due to steric considerations. Table 2 includes the values obtained for the adsorption of the PCDD series on the SWCN.

Initially, the reactions were modeled for a small set of PCDDs outside (on top) of the SWCN curved surface, where the initial state of the reaction is each PCDD (described in Table 1) and two H atoms. The reaction pathways were explored for OCDD through all possible routes to the dehydrohalogenated products, Pe_4_CDD, T_1_CDD and TrCDD. As expected, each resulting PCDD that was formed was accompanied by the formation of a HCl molecule.

**Figure 6:**
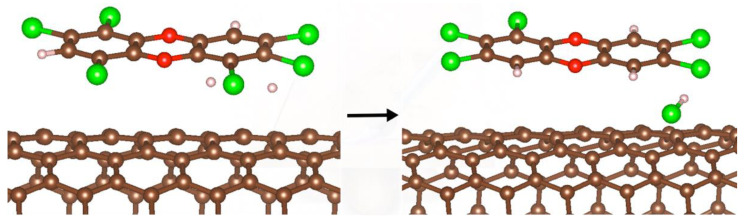
Hydrodechlorination of a HxCDD obtaining a PCDD and a HCl molecule. The brown spheres represent C atoms, the green spheres represent Cl atoms and white spheres are H atoms.

For instance, the reaction between the adsorbed Hx_7_CDD on the SWCN model with two hydrogen atoms led to obtaining a HCl molecule and the adsorbed PeCDD on the SWCN (see Figure 6).

Hx_7_CDD/SWCN + 2 H → HCl + PeCDD/SWCN

Considering the example described above, the hydrodechlorination energy of the Hx_7_CDD was obtained according to the following expression:

E_rxn_ = E_PeCDD+HCl/SWCN_ − (E_Hx7CDD_ − E_SWCN_ + E_2H_)

The same procedure was used for the dehydrochlorination of the PCDD series adsorbed on the SWCN model.

**Figure 7:**
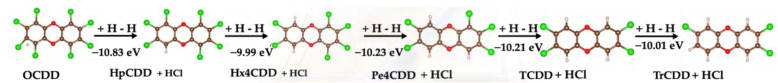
Full consecutive dehydrogenation of the set of PCDDs, from the octachlorodibenzodioxin molecule to trichlorodibenzodioxin. The brown spheres represent C atoms, the green spheres represent Cl atoms, the red spheres represent O atoms and white spheres are H atoms.

Table 2 shows the reactants and products of the hydrodechlorination of each dioxin, the adsorption energy of the dioxin that will be dehydrochlorinated and the calculated reaction energy (E_rxn_) of each dioxin adsorbed on the SWCN plus two hydrogen atoms, as reactants; it also shows the distances between the two products, namely, the partially dehydrogenated PCDDs and HCl molecules; and Figure 7 presents the full dehydrochlorination of the set of PCDDs, from the OCDD to TrCDD.

The calculated bond distance of the Cl-C bond within a PCDD molecule is 1.71 Å. After the reaction with hydrogens took place, it was observed that one of the chlorine atoms was replaced by a H atom forming a H-C bond and a hydrochloric acid molecule was eliminated. The C-Cl bond length compares very well with the bond distance of other organic compounds. The final distance between the reacting chlorine atom and the carbon atom to which it was bonded was 4.18 Å in the different systems studied. This can be interpreted as a chlorine atom totally separated from the dioxin molecule. The atomic H-C distance in the newly formed bond of the dioxin was found to be 1.09 Å long, whereas the H-Cl distance was calculated at 1.29 Å. The calculated reaction energy between −9.99 eV and −10.83 eV indicates that the reactions were exothermic.

Let us give a brief annotation about the three isomers of the hexachlorodibenzodioxin: the dehydrochlorination energy is identical, and in all cases pentachlorodibenzodioxin is obtained. On one hand, through the dehydrochlorination of Hx_4_CDD (1,2,3,4,7,8-HxCDD), Pe_4_CDD (1,2,4,7,8-PeCDD) is obtained; on the other hand, the dehydrochlorination of Hx_6_CDD (1,2,3,3,6,7,8-Hexachlorodibenzodioxin) and Hx_7_CDD (1,2,3,7,8,9-Hexachlorodibenzodioxin) yields 1,2,3,7,8-PeCDD.

### 2.6. Dehydrochlorination of Polychlorodioxins on a SWCN with Ni_x_ (x = 10, 13) Clusters and H_2_

The dissociation of molecular hydrogen is an endothermic process requiring a great deal of energy, i.e., 4.5 eV; this process cannot take place by itself on a carbon nanotube. In contrast, we have shown that H_2_ dissociation is plausible on the upper edges or the vertex of nickel clusters, namely, N_10_ and N_13_. The dissociation energies for H_2_ on the aforementioned metal systems was calculated to be around −5.5 eV, as indicated earlier. Furthermore, we have also shown that atomic hydrogen readily reacts with dioxins leading to a substitution of a chlorine atom by an atomic hydrogen atom, and to the formation of a HCl molecule. Moreover, the adsorption energy of the nickel clusters onto a carbon nanotube is significant at −3.34 eV, thus these findings suggest that a process involving the dehydrohalogenation of dioxins is feasible if molecular hydrogen is placed in an environment where nickel clusters and SWCNs are present.

## 3. Materials and Methods

All calculations were performed within the computational package VASP (v5.3.3) [74,75,76] and models were visualized with the VESTA software [77]. Spin-polarized DFT calculations were performed using the PAW method with the Grimme [70] exchange-correlation functional and the generalized gradient approximation (GGA) modified to consider the dispersion effects. All structures were fully optimized until the total energy converged to within 10^−6^ eV during the self-consistent calculations when forces converged to 0.02 eV/Å. An energy cut-off of 450 eV was used. Only one gamma-point sampling in the first Brillouin zone was used for all calculations. The Methfessel–Paxton method with a smearing of 0.2 eV was used for the metal adsorption to the SWCN and the Gaussian smearing method for molecule systems.

For the SWCN and dioxin investigation, both the SWCN and PCDD molecules were built with Materials Studio 4.4 [78], with unit cell measurements of 26.6 × 21.7 × 19.5 Å containing a total of 344 atoms. The armchair structure for the SWCN was chosen in this study, and the internal diameter of the SWCN constructed was 13.75 Å.

To identify each PCDD molecule, codes were assigned to each one as shown in Table 1. Additionally, for the optimized calculations and reactions, the SWCN, PCDD, and 2 H atom systems were also assigned codes for identification, as shown in Table 2.

The reactions investigated consisted of SWCN + TCDD + 2 hydrogen atoms. For these systems involving all the dioxins listed in Table 1, the following energy equation was used to determine the reaction energy, E_rxn_:

E_rxn_ = E_PCDD+H-H/SWCN_ − (E_SWCN_ + E_PCDD_ + E_H-H_)

where E_rxn_ is the reaction energy of the PCDD molecule and the hydrogen atoms on the nanotube. E_SWCN_ is the energy of the nanotube, E_PCDD_ is the energy of the corresponding PCDD molecule isolated, and E_H-H_ is the energy of the hydrogen atoms.

The vibrational frequencies of the products were calculated and found to be positive for all states. The imaginary frequencies found were identified to be those associated with the HCl molecules and with a non-significant value. No further refinements were carried out, as it was assumed that all the states from the SWCN and dioxins were stationary.

## 4. Conclusions

Dihydrogen dissociation on a SWCN is difficult at best; however, the literature references demonstrated that metal atoms supported on carbon nanotubes are more suitable for dihydrogen dissociation, which makes H atoms available for reactions. Taking into account the latter, we investigated dihydrogen dissociation onto Ni_13_ and Ni_10_ clusters and found that both clusters are suitable for H_2_ dissociation. Furthermore, dioxins adsorbed on a carbon nanotube do not react with H_2_, yet, we have shown that free H atoms can dehydrohalogenate a dioxin with energies which were similar for all of the dioxins investigated and with comparable energies for all the C-Cl substitutions. Thus, we can conceive that nickel clusters physisorbed onto carbon nanotubes can readily cleave H_2_ into atomic hydrogens which can attack and substitute a chlorine atom in a carbon nanotube-adsorbed dioxin molecule. For each H_2_ molecule, a H-Cl substitution would take place while also giving HCl as a byproduct.

## Figures and Tables

**Table 1:** Description of the codes used for the PCDD series of this study.

Code	PCDD Abbreviation	IUPAC Nomenclature
OCDD	OCDD	Octachlorodibenzodioxin
HpCDD	1,2,3,4,6,7,8-HpCDD	1,2,3,4,6,7,8-Heptachlorodibenzodioxin
Hx_4_CDD	1,2,3,4,7,8-HxCDD	1,2,3,4,7,8-Hexachlorodibenzodioxin
Hx_6_CDD	1,2,3,6,7,8-HxCDD	1,2,3,6,7,8-Hexachlorodibenzodioxin
Hx_7_CDD	1,2,3,7,8,9-HxCDD	1,2,3,7,8,9-Hexachlorodibenzodioxin
PeCDD	1,2,3,7,8-PeCDD	1,2,3,7,8-Pentachlorodibenzodioxin
Pe_4_CDD	1,2,4,7,8-PeCDD	1,2,4,7,8-Pentachlorodibenzodioxin
TCDD	2,3,7,8-TCDD	2,3,7,8-Tetrachlorodibenzodioxin
T_1_CDD	1,3,7,8-TCDD	1,3,7,8-Tetrachlorodibenzodioxin
TrCDD	2,3,7-TrCDD	2,3,7-Trichlorodibenzodioxin

**Table 2:** Energies for the adsorption and reactions modeled on the SWCN model, including the final distance between the dehydrogenated product PCDD and a HCl molecule.

Reactants	Products	d_PCDD–HCl_ (Å)	E_ads_ (eV)	E_rxn_ (eV)
OCDD/SWCN + H-H	HpCDD/SWCN + HCl	3.27	−0.60	−10.83
HpCDD/SWCN + H-H	Hx_4_CDD/SWCN + HCl	3.99	−0.41	−9.99
HpCDD/SWCN + H-H	Hx_6_CDD/SWCN + HCl	4.08		−9.99
HpCDD/SWCN + H-H	Hx_7_CDD/SWCN + HCl	4.07		−9.99
Hx_4_CDD/SWCN + H-H	Pe_4_CDD/SWCN + H-Cl	3.97	−1.10	−10.23
Hx_6_CDD/SWCN + H-H	PeCDD/SWCN + H-Cl	4.03	−1.10	−10.20
Hx_7_CDD/SWCN + H-H	PeCDD/SWCN + H-Cl	4.01	−1.10	−10.20
PeCDD/SWCN + H-H	TCDD/SWCN + H-Cl	3.98	−1.05	−10.21
TCDD/SWCN + H-H	TrCDD/SWCN + H-Cl	4.22	−0.96	−10.01

## Data Availability

Not applicable.
